# Adhesive Bioinspired Coating for Enhancing Glass-Ceramics Scaffolds Bioactivity

**DOI:** 10.3390/ma15228080

**Published:** 2022-11-15

**Authors:** Devis Bellucci, Annachiara Scalzone, Ana Marina Ferreira, Valeria Cannillo, Piergiorgio Gentile

**Affiliations:** 1 Dipartimento di Ingegneria “Enzo Ferrari”, Università Degli Studi di Modena e Reggio Emilia, Via P. Vivarelli 10, 41125 Modena, Italy; 2 School of Engineering, Newcastle University, Stephenson Building, Claremont Road, Newcastle upon Tyne NE1 7RU, UK; 3 Center for Advanced Biomaterials for Health Care@CRIB Istituto Italiano di Tecnologia, Largo Barsanti e Matteucci 53, 80125 Napoli, Italy

**Keywords:** surface functionalisation, nano-coating, glass-ceramics, porous scaffold, bone regeneration

## Abstract

Bioceramic scaffolds, composed of a biphasic composite containing bioactive glass and hydroxyapatite, were prepared in this work to overcome the intrinsic limits of the two components taken separately (in particular, their specific reactivities and dissolution rates, which should be tunable as a function of the given clinical requirements). To mimic the biological environment and tune the different stages of cellular response, a coating with gelatin and chondroitin sulphate via Layer-by-Layer (LbL) assembly was presented and discussed. The resulting functionalized scaffolds were affected by the coating in terms of microstructure and porosity. In addition, the LbL coating significantly enhanced the seeded cell behaviour, with high adhesion, proliferation and osteogenic activity, as revealed by the alkaline phosphatase activity and overexpression of osteopontin and osteocalcin.

## 1. Introduction

A fundamental step in bone tissue engineering is the development of porous structures as supporting scaffolds, which aid seeded cells to produce new tissue [1,2]. To best perform their function as a temporary extracellular matrix (ECM), scaffolds must have an architecture that ideally reproduces that of the tissue to be regenerated. In the case of bone, this architecture provides for a compact and hard outer shell (cortical bone) that protects a spongy internal area (trabecular bone) [3]. The ultimate goal of scaffolds is, on the one hand, to ensure adequate mechanical support for the cells until they have produced their ECM; on the other hand, to let the nutrients supply and the disposal of waste products of cellular metabolism. In this way, it is possible to guide the behaviour of the cells, by reproducing an environment favouring cell adhesion, proliferation and differentiation. Therefore, the scaffolds must have the right degree of permeability, which means a porosity of the order of 80/90% with pores of at least 100 μm to facilitate cell infiltration and migration [4]. Furthermore, the degradation rate of the scaffold, which is gradually replaced by the ECM and by the new tissue, must be adequate not to compromise the necessary mechanical support.

Ceramic materials such as hydroxyapatite (HA) or bioactive glasses are among the most used in the field of regeneration and repair of bone tissues [1,2,3]. In fact, HA is already one of the main components of bone, to which it confers rigidity and hardness [3]. However, it has the defect of not being reabsorbed once implanted in the human body. Bioactive glasses, on the other hand, are more reactive than HA in a physiological environment and can rapidly bind to bone tissue [5]; used in the form of granules, they favour tissue regeneration and repair. In recent years, many bioactive glasses have been developed and the relationship between their specific chemical composition and reactivity has been studied [6]. An interesting alternative is to create biphasic composites containing bioglasses and HA, to overcome the limits of the two components taken separately [7]. In this way, on the one hand it is possible to modulate the bioactivity and resorbability of these systems by modifying the volumetric fractions of the components in an appropriate manner; alternatively, the glass phase acts as a sintering aid, aiming to favor the composite densification and its mechanical strength. Moreover, when silicate bioactive glasses are employed in HA-based composites, favorable ionic substitutions may occur in the apatite lattice [7]. However, as the manufacturing of these composites involves a heat treatment to sinter the powders, it is crucial that (1) the glass used does not crystallise before sintering and (2) the temperature of the heat treatment is low enough to not induce decomposition of HA with the development of unwanted phases and/or the reaction between HA and the glass itself [7]. In fact, the crystallisation is reported to slow down the glasses’ reactivity and ion release, thus limiting the biological performance of these materials [8,9]. Therefore, bioglasses with a high crystallisation temperature are particularly interesting in this context, such as BG_CaMix (composition in mol%: 2.3 K_2_O, 2.3 Na_2_O, 45.6 CaO, 2.6 P_2_O_5_ and 47.3 SiO_2_), which has a crystallisation temperature of about 880 °C [9]. As a comparison, 45S5 Bioglass^®^, the first bioactive glass produced and the most widely used, crystallises at temperatures of the order of 600 °C [8,10]. Thanks to the use of BG_CaMix, it is possible to successfully create biphasic composites with HA as a second phase, keeping the glass phase substantially amorphous after the sintering process [11].

In this work, a BG_CaMix/HA composite is used to make scaffolds by combining a modified foam replication protocol with a polymer burning out technique [12]. The result is a scaffold with an inner highly porous structure and an outer more compact and permeable shell; this would ensure adequate handling of the synthetic graft and excellent mechanical support for the growing tissue.

Furthermore, in this work we proposed to bio-functionalise the scaffolds to mimic the biologically relevant environment and modulate the different stages of cellular response [13]. Indeed, since cell-material interaction is established between the binding-receptors on the cell surface and binding-sites present in proteins of the ECM, biomolecules and adhesion proteins present in the extracellular matrix (e.g., collagen, fibronectin, etc.) are commonly used in the bio-activation of material surfaces [14,15]. In former studies, we have investigated the adhesive properties of a dopamine-conjugated chondroitin sulphate (CS-DP) as coating for different substrates, such as PLA, PCL, TiO_2_, and SiO_2_ [16]. We observed that, following the coating, the surfaces showed high hydrophilicity (<40° of contact angle). In addition, the polymer demonstrated to be cytocompatible, by enabling adhesion, proliferation and ECM production of immortalized TERT human mesenchymal stem cells (Y201) onto PLA substrates.

Therefore, in this work, BG_CaMix/HA composite porous scaffolds have been functionalised by Layer-by-Layer assembly [17,18] with gelatin (as positive-charged polyelectrolyte, polycation) and CS-DP (as negative-charged polyelectrolyte, polyanion) to study Y201 behaviour. The influence of the porous structure of the scaffold and the molecular topography created by gelatin/CS-DP nanocoating over Y201 cell adhesion and differentiation has been evaluated. This work represents the first study where the bioactive properties of the gelatin are combined with the tissue adhesive properties of catechol-based chondroitin sulphate for improving the biological behavior of mesenchymal stem cells, particularly the osteoinductivity to enhance their differentiation in osteoblasts with the formation of new bone tissue.

## 2. Materials and Methods

### 2.1. Materials

Sodium chloride, *N*-(3 Dimethylaminopropyl)-*N*-ethylcarbodiimide hydrochloride and N-Hydroxysuccinimide were purchased from Sigma-Aldrich (Gillingham, UK). The distilled water was obtained with a Milli-Q Integral system equipped with a BioPak ultrafiltration cartridge (Millipore, Merck, UK).

Raw powders for the production of BG_CaMix was purchased from Carlo Erba Reagenti (Milan, Italy); HA (CAPTAL^®^ Hydroxylapatite, Plasma Biotal Ltd., Tideswell, UK) was used to prepare BG_CaMix/HA composites.

### 2.2. BG_CaMix/HA Composites Preparation

Commercial raw powders were used to prepare the BG_CaMix glass by means of a melting-quenching protocol, as previously described in Refs [9,19]. Raw materials powders were carefully mixed for 8 h in a plastic vessel rolling on a shaker and subsequently melted in a platinum crucible at 1450 °C for 1 h. The BG_CaMix melt was quenched in room temperature water to obtain a glass frit, which was left to dry in a furnace at 110 °C for 24 h. The frit was ground in a porcelain jar for 40 min and then sieved to produce a powder (grain size < 45 μm), which was subsequently add to HA powders to obtain the BG_CaMix/HA composite. Commercial HA (particle size below 25 μm) and BG_CaMix were mixed for 8 h in a plastic can using a roll shaker in order to prepare a composite with the following composition: 70 wt.% BG_CaMix and 30 wt.% HA powders.

### 2.3. Scaffold Fabrication

A modified foam replication method has been employed to prepare highly porous scaffolds, as described in detail elsewhere [20,21]. Briefly, BG_CaMix/HA powders were used to produce a slurry, together with distilled water, a polyvinylic binder (which was employed to favour the adhesion of the slurry to the sponges) and polyethylene powders, added as an additional porogen. Then, 50 wt.% distilled water, 35 wt.% BG_CaMix/HA powders, 10 wt.% polyvinylic binder and 5 wt.% polyethylene powder (Goonvean Fibres, Cullompton, UK) were properly mixed in a beaker for 30 min under magnetic stirring. Polyurethane sponges (2 cm × 2 cm × 1.5 cm) were employed as templates to produce samples with the desired shape and porosity The sponges were dipped into the slurry, properly impregnated, and retrieved without squeezing; for these reasons, the impregnation procedure was different than that typically used in the traditional replication [22,23], where impregnated sponges are carefully squeezed before drying and dry very slowly. Herein, on the contrary, the samples (loaded with the slurry) were rapidly dried in a hot air flux; moreover, polyethylene particles were added to the slurry, as in the standard polymer burning-out protocol [24], with the aim of further increasing the porosity of the scaffolds resulting from their spongy architecture (in particular at their surfaces, which act as porous “shells”, thus making the samples easy to handle but, at the same time, higly permeable to fluids and cells). Finally, the samples were sintered at 820 °C for 3 h (heating rate: 3 °C/min up to 500 °C and then 5 °C/min).

### 2.4. Layer-by-Layer Assembly

The coating of the glass-ceramic scaffolds with a multilayered coating was performed at room temperature. The conjugated chondroitin sulphate (Chondroitin 4-sulfate sodium salt obtained from bovine trachea (CS, CAS No: 39455-18-0)) with dopamine hydrochloride (DP, CAS No: 62-31-7) (CS-DP) was synthetised as reported by the authors previously [16]. Both gelatin and CS-DP polyelectrolytes were dissolved at 2% *w*/*v* in 0.1 M NaCl (pH~6.5; 5 mg/mL). Laser Doppler electrophoresis (Zetasizer Nano, Malvern instrument, Enigma Park, Great Malvern, UK) was used to measure the ζ-potentials of the polyelectrolyte solutions priorly the LbL assembly procedure. Firstly, the scaffolds were immersed in a gelatin solution, made by dissolving 5 mL of gelatin (from porcine skin—Type A powder) for 15 min. Subsequently, samples were washed for 5 min in water containing 0.1 M NaCl (pH~6.5), prior to be soaked in CS-DP solution (5 mL) for 15 min followed by a water washing step, using the same parameters described before. Such immersion and washing procedure was repeated for 4 cycles to obtain 8 layers. Then, the coated scaffolds were soaked in a genipin (CAS No: 6902-77-8) solution characterised by a concentration of 5% *w*/*v* in distilled water to crosslink the coating and thus increase the stability. Uncrosslinked nanocoated scaffolds were also prepared and used as control. Finally, all the coated scaffolds were left to dry overnight and properly stored at 4 °C.

### 2.5. Physico-Chemical Characterisation of the Functionalised Coating on Composite Scaffolds

Scaffold’s morphology was observed with a Scanning electron microscope (SEM, Hitachi TM3030, Krefeld, Germany).

Infrared spectroscopy (FTIR-ATR) analysis was performed on samples. The infrared spectra were acquired with a spectrophotometer equipped with ATR sampling accessory (UATR Spectrum One, Perkin Elmer, Beaconsfield, UK). The readings were taken in 4000–550 cm^−^^1^ wavelength range of with 16 scans and resolution of cm^−^^1^.

X-ray photoelectron (XPS) spectra were obtained by means of a Theta Probe (Thermo Scientific, Oxford, UK) equipment with a microfocused AlKa X-ray source (1486.6 eV) and operated with spot size of 400 µm. The following parameters were used: 200 eV pass energy, 1 eV step size, 50 ms dwell time in not angle-resolved lens mode. On each scaffold surface, at least 3 single area were investigated.

### 2.6. Biological Characterisation of the Functionalised Coating on Composite Scaffolds

#### 2.6.1. Cells Culture and Seeding

In vitro cell tests were performed on the porous scaffolds before and after LbL functionalization. Before seeding the cells, UV light was used to sterilize the scaffolds for 4 h in 12-well untreated plates and rinsed 5 times with PBS (pH 7.4). Human TERT immortalised bone marrow stromal cells (Y201—supplied by Prof P. Genever, York University, UK) at passage 80 and cultured at 37 °C and 5% CO_2_ in Dulbecco’s Modified Eagle Medium (DMEM) with low glucose content, supplemented with 10% Foetal bovine serum (FBS), 2 mM L-glutamine and a 1% Penicillin/Streptomycin (P/S). Such medium conditions are considered as basal. Then, 100,000 cells were seeded onto the scaffolds in 5 mL DMEM.

#### 2.6.2. Cell Viability Using Human Bone Marrow-Derived Mesenchymal Stem Cells (hMSCs)

The culture medium was removed after 3 and 7 days of cell culture and the samples were transferred to new 12-well plates; subsequently, 10% PrestoBlue solution was added (5 mg/mL in DMEM; Thermo Scientific, Oxford, UK) and the multi-well plates were incubated at 37 °C for 2 h. After the supernatant removal, the solution was transferred in 96-well plates (0.2 mL) and quantified with a spectrophotometer working at 560 nm; a Filter-based FLUOstar^®^ Omega multi-mode reader (FLUOstar^®^ Omega, BMG Labtech, Ortenberg, Germany) was used. PicoGreen^®^ dsDNA reagent purchased from Invitrogen (Carlsbad, CA, USA) was employed to calculate the number of the cells for each sample to make a correct normalisation of the fluorescence values. The scaffolds were carefully washed with PBS after each culturing period, incubated for 3 h at 37 °C for 3 h and then frozen at −80 °C overnight in ultra-pure water (1 mL) in order to ensure cell lysis. The assay was carried on according to the protocol of the manufacturer. Fluorescence was measured at an excitation and emission wavelength of 485 and 528 nm, respectively.

#### 2.6.3. Alkaline Phosphatase Activity (ALP)

ALP was investigated after 7, 14 and 21 days. To this aim, alkaline buffer solution (500 µL) and stock substrate solution (0.5 mL, 40 mg p-nitrophenyl phosphate disodium, Sigma-Aldrich, Gillingham, UK) were added to 100 µL of each lysate sample (resulting from the procedure previously reported for the PicoGreen assay), diluted in 10 mL of distilled H_2_O at 37 °C for 1 h. The p-nitrophenol production was analyzed by evaluating the solution absorbance (a Leica DM2500 equipment working at 410 nm was employed). PicoGreen^®^ dsDNA reagent, purchased from Invitrogen (Waltham, MA, USA), was used to estimate the number of cells for each scaffold after normalization with the ALP values of absorbance.

#### 2.6.4. Osteocalcin and Osteopontin Detection

Osteocalcin (OC) and osteopontin (OP) and protein expression of Y201 was investigated by immunoassay method, aiming to evaluate the osteoblast differentiation. OC and OP concentration was determined using the lysates used for DNA quantification by Picogreen. OP quantitative estimation was carried on employing a Mouse/Rat Osteopontin Quantikine ELISA Kit (R&D Systems, Minneapolis, MN, USA). Briefly, 50 µL of assay diluent RD1W and 50 µL of standard (2500 to 39 pg/mL) were added to control and scaffolds in the multi-well plate and incubated for 2 h at 25 °C. After 4 washing steps, 100 µL of Mouse/Rat OPN conjugated were added and then further incubated for 2 h at 25 °C. Before adding 100 µL of stopping solution, the sandwich complex was rinsed 4 times to react with 100 µL of substrate solution. Finally, the optical density was determined at 450 nm and concentration of OP obtained from standard curve plot. OC quantitative measurement was performed by means of a Rat Bla-Osteocalcin High Sensitive EIA kit (Takara Clontech, Shiga, Japan). In this case, 100 µL of samples and standard solution (16 to 0.25 ng/mL) were properly incubated at 37 °C for 1 h with the capture-antibody, rat osteocalcin C-terminus-specific antibody. After OC capture and 3 washing steps, 100 µL of the enzyme-labelled antibody (GlaOC4-30) specific to Gla-OC was incubated for 1 h at room temperature. The sandwich complex was washed 4 times and allowed to react with 100 µL of substrate solution for 10–15 min. After adding the stop solution, the optical density was measured at 450 nm and the concentration of the OC obtained from standard curve was plotted. OC and OP content was calculated after normalization of the concentration of OC or OP per DNA concentration considering each time point and condition.

#### 2.6.5. SEM Analysis

The surface of coated scaffolds, seeded with cells, was observed in a scanning electron microscope (ESEM Quanta 2000, FEI Co., Eindhoven, The Netherlands). Prior to observation, the samples were sputter-coated with Au.

### 2.7. Statistical Analysis

Tests were performed at least 3 times for each scaffold. All data were expressed as mean ± SD. Graph pad Prism 6 software (version 6, Dotmatics, San Diego, CA, USA) was used for statistical analysis. Kruskal-Wallis One Way Analysis of Variance on Ranks (ANOVA) was employed to calculate the statistical differences between groups. Statistical significance was declared at * *p* < 0.05, ** *p* < 0.001 and *** *p* < 0.0001.

## 3. Results and Discussion

Both the BG_CaMix/HA composite and the uncoated scaffolds here presented have been previously characterized [11]. Their main properties are:

- Despite the sintering temperature is nominally lower than the crystallisation temperature of BG_CaMix [9], the glass phase in the composite scaffolds underwent a partial crystallisation, with the formation of wollastonite (CaSiO_3_); this is reasonable, considering the 3 h isothermal step performed to assist the sintering process. On the other hand, the possible development of wollastonite is expected to maintain the ability of the samples to bond to bone tissue, as wollastonite is reported to be highly bioactive [25,26];
- The obtained BG_CaMix/HA scaffolds displayed a highly porous internal network (with a porosity of about 85 vol.%), with strong trabeculae and large pores, coupled to an outer permeable “shell”, that ensures good manageability;
- The bioactivity of the scaffolds has been successfully verified in vitro by soaking the samples in a simulated body fluid solution (SBF), as recommended by the literature [27].

**Figure 1:**
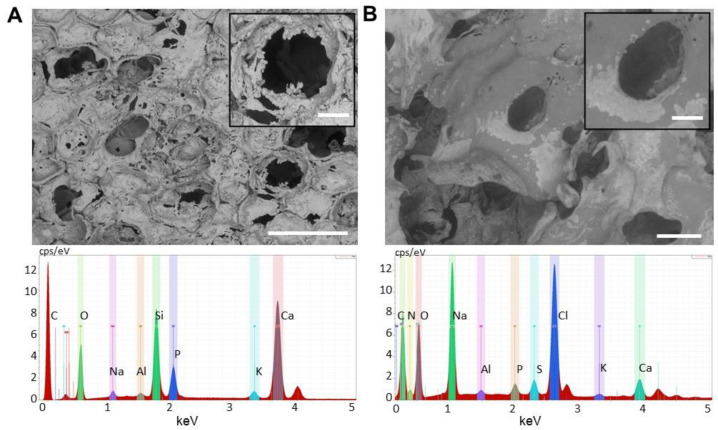
SEM micrographs (on the top, bar = 1 mm) and EDS analysis (on the bottom) of the 3D porous glass-ceramic scaffold before (**A**) and after Layer-by-layer surface modification (**B**). The insets of the SEM show the macrographs of the pores (Magnification 200×, bar = 200 μm).

For mimicking the bone structure and morphology, LbL approach was used for obtaining a multilayered coating on the glass-ceramic porous scaffolds to incorporate biomimetic macromolecules into the nanolayers (Figure 1). Furthermore, 8 layers were selected as final number after optimization of several process parameters: number of layers (6, 8 and 10), dipping time into both polyelectrolytes (5 and 15 min), PE concentration (2, 5 and 10 mg/mL), genipin concentration (2.5 and 5%) and disposition of the nanolayers (gelatin or CS-DP as final top layer). Gelatin and chondroitin sulfate were selected since these are natural polymers. Gelatin resembles its precursor collagen, which is one of the most abundant components of the extracellular matrix; while chondroitin sulfate is a proteoglycan present in several tissues of the human body, where plays an important role in regulating cell functions. Both polymers allow for increased cell adhesion, differentiation and proliferation [28].

Before the LbL assembly, the conjugation of dopamine to the chondroitin sulphate did not affect the ζ-potential of the PE solution (−16.3 mV respect with −18.9 mV of pure CS), while the gelatin solution was always positively charged with ζ-potential of +10.8 mV.

The surface morphology of the glass-ceramic scaffolds before and after LbL assembly was analysed by SEM (Figure 1). The 3D porous scaffolds presented an average pores diameter of 355 ± 125 µm, suitable for the cells migration and proliferation and allow new bone formation [29]. Furthermore, small porosity with an average diameter of 12.4 ± 3.1 µm were observed and appropriate for nutrient diffusion, making accessible biocues of the native ECM [30].

After the LbL assembly, significant differences in terms of porosity have been detected (both in terms of pore size and total porosity). Also, a smooth coating could be easily observed, compared to the roughness of the scaffold surface Figure 1B.

Successful immobilisation of gelatin and chondroitin sulphate-conjugated with dopamine was monitored by several techniques. Specifically, EDS proved the formation of the multilayer coating by the amount of sulphur and nitrogen calculated. For the coated scaffolds, S and N content was 6.2 ± 0.2 wt.% and 3.5 ± 0.4 wt.% considerable higher than the amounts detected in the uncoated scaffolds (0 wt.% for sulphur and 0.1 wt.% for nitrogen).

**Figure 2:**
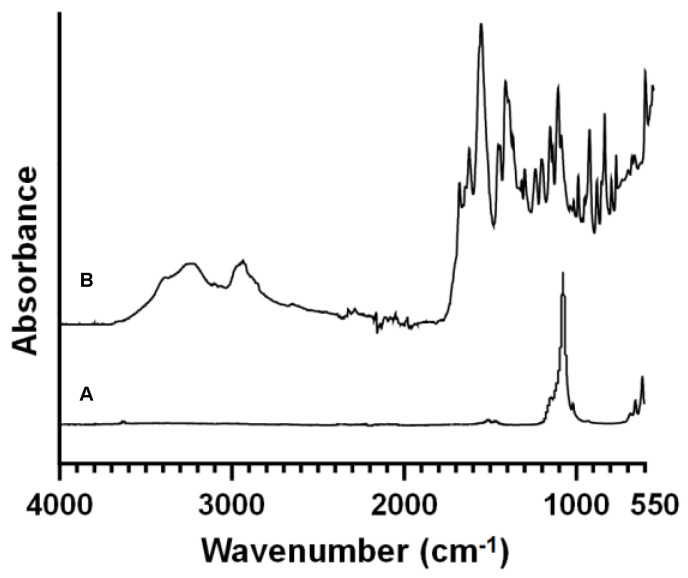
ATR-FTIR spectra of the 3D porous glass-ceramic scaffold before (**A**) and after Layer−by−layer surface modification (**B**). Resolution = 4 cm^−1^.

Moreover, XPS and ATR-FTIR were carried on in order to investigate the surface composition of the membranes before and after LbL assembly. In particular, the infrared spectra showed in Figure 2A of the uncoated scaffold shows the typical peaks of Si-O-Si symmetric and asymmetric stretching vibration at 802 and 1086 cm^−1^, respectively, and the peak at 950 cm^−1^, which corresponds to the P-O stretching vibration. The peaks at 1635 and 3450 cm^−1^ are ascribable to the O-H bonds and indicate the water trapped inside the sample. Finally, the narrow band near 1384 cm^−1^ is attributable to the carbonate group (CO_3_^2−^).

After the LbL functionalisation, Figure 2B reveals the presence of the typical bands of the polyelectrolytes: for the gelatin the characteristic absorption bands at 1630 and 1529 cm^−1^, ascribable to amide I and amide II, as well as the absorption band at 1238 cm^−1^, related to amide III. The broad absorption band centered at 3288 cm^−1^ was referred to –OH and –NH (amide A) stretching vibrations) [31]. CS-DP was characterised by the following chemical bands: Methylene groups (–CH_2_), observed at 2940–2920 and 2860–2850 cm^–1^, and the amide C=O stretching signals in the 1700–1250 cm^–1^ interval. At 1650–1500 cm^–1^ Carboxylate moieties (COO–) belonging to salts were detected, while the NH bending signal was observed at 1560–1530 cm^–1^. The primary –OH-stretch occurred at 3640–3630 cm^–1^, beyond the signal at 3350–3250 cm^–1^. Finally, Ethers’ contribution (–C–O–C–) was recorded at 1100 cm^–1^ [16]. No significant differences were detected between the coated scaffolds with and without the crosslinking of genipin as also described by Tonda-Turo et al. [32] and Panzavolta et al. [33].

**Figure 3:**
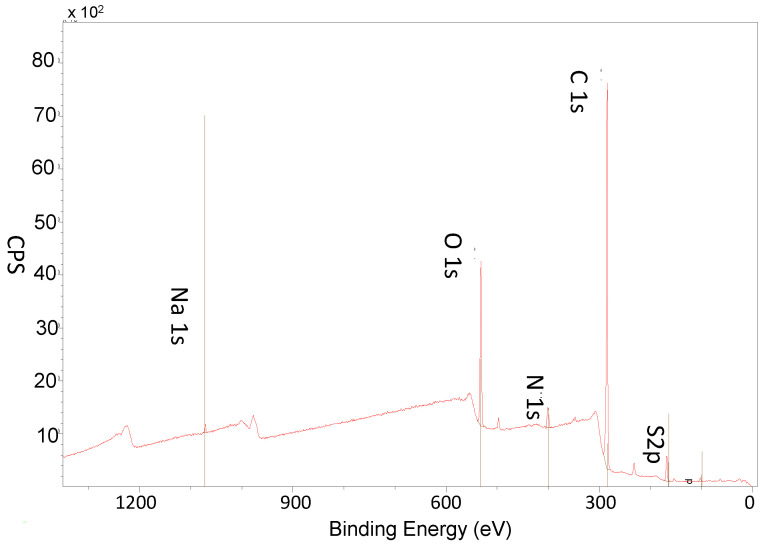
XPS survey spectra of the functionalised porous scaffold after the obtainment of 8 nanolayers.

Figure 3 shows the XPS survey spectra after the obtainment of 8 nanolayers, evidencing the representative N1s peak (399.5 eV) and S2p peak (168 eV), demonstrating the CS-DP and gelatin have been successfully introduced. Table 1 reports the atomic percentage of the representative coating elements (C1s, N1s, O1s and S2p), together with the calculated atomic ratio of S/N by increasing the number of layers.

XPS results confirmed the obtainment of the multilayers, showing an increase if the content of sulphur and nitrogen. Furthermore, the atomic ratio of S/N showed an alternating consistent pattern, thus suggesting specific changes on the chemical composition of the surface after the LbL functionalisation. In particular, the S/N ratio presented an higher value when the top-layer was CS-DP where sulphur was the representative chemical element (0.33 ± 0.11 after 8-layer coating compared with 0.10 ± 0.12 after 7-layer coating with gelatin as top layer). Although XPS can be used for the evaluation of atomic concentrations of the elements presented in thin films or coatings using the areas of selective peaks, the reported values need to be considered as semi-quantitative because the area determination can be affected by several factors including the background correction, overlapping peaks, multiplet splitting, shaker/satellite peaks, etc.

The design and manufacture of biocompatible materials is key challenge for in vivo implantation of 3D porous scaffolds. Thus, different strategies have been reported in literature for successfully modifying scaffold surfaces with coating, like with chemical treatment, physical absorption, ionic encapsulation [34,35]. However, physical absorption and encapsulation can provide a very weak stability of the surface coatings because they degrade quite quickly and not addressing the requirement of functionalised scaffolds that need to be implanted for medium/long-term.

In this work, we selected the LbL assembly, due to its environmental-friendly and versatile nature, largely proposed in different applications, and it permits to create easily a nanocoating with biomacromolecules to get precise biological activities. Furthermore, in this research the covalent crosslinking with the genipin allowed to avoid a quick release of the nanocoating, due to the hydrolytic processes happening in physiological solutions. Similar approach has been proposed by Mano et al., who prepared chitosan (CHIT)/alginate (ALG) freestanding membranes fabricated by layer-by-layer assembly and, then, cross-linked with genipin. This crosslinking led to an increase of storage modulus, Young modulus, and ultimate tensile strength, but to a decrease in the maximum hydrostatic pressure [36].

**Figure 4:**
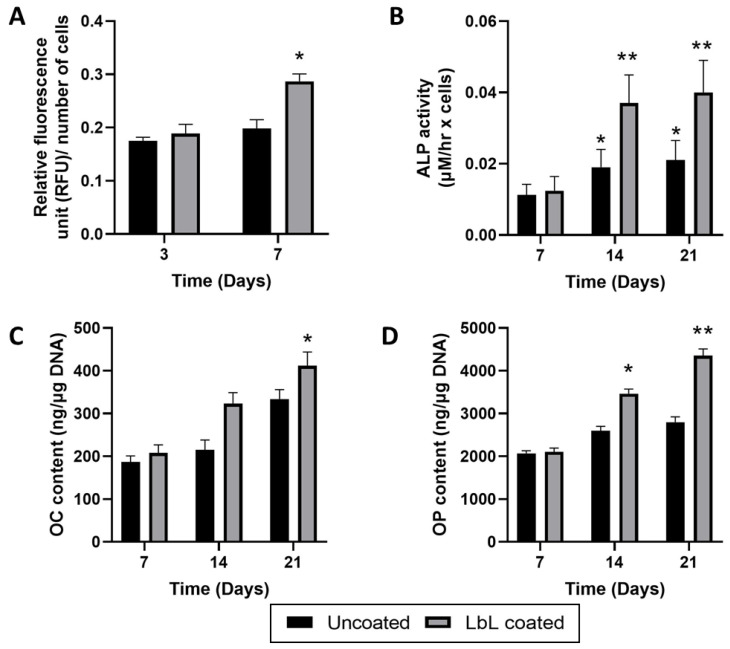
In vitro cell tests. (**A**) Y201 metabolic activity (PrestoBlue^®^ assay) after culturing for 3 and 7 days. (**B**) Alkaline phosphatase activity, (**C**) Osteopontin protein content and (**D**) Osteocalcin protein content of Y201 at 7, 14 and 21 days. Statistics: * *p* < 0.05 and ** *p* < 0.001.

The design of the functionalised porous scaffolds here presented was proposed after combination of several process variables and taking into account both physico-chemical characterisation and investigating cells behaviour. In this study we used Human TERT immortalised bone marrow stromal cells (Y201), that can represent adult stem cell population able to differentiate into various lineages [37]. Thus, in vitro cell tests were performed to investigate if the selected combination of the biopolymers as polyelectrolytes and the chemical crosslinking with genipin influenced the Y201s viability and proliferation. PrestoBlue assay (Figure 4A) presented that Y201s in both uncoated and coated scaffolds showed a similar metabolic activity after 3 days of culture while, after 7 days, onto the coated samples Y201s showed an increased significant metabolic activity (normalised RFU for uncoated and coated were 0.198 ± 0.017 and 0.287 ± 0.014 respectively).

Therefore, the addition of nanocoating influenced intensely the adhesion and metabolic activity of Y201: in particular, we found that the cells were more viable on scaffolds with the tissue-adhesive CS-DP as the top layer, compared with GEL (data not shown). Scalzone et al. also described the ability of the CSDP hydrogel to support Y201 cells viability and metabolic activity; whereas the CS-DP formulation as bio-adhesive showed to improve considerably the hydrophilicity properties of different substrates [16]. Furthermore, the selection of polyelectrolyte as top layer can influence strongly the cellular behaviour. In fact, Ferreira-Duarte et al. reported that the number of deposited heparin layers as top-layer (1 or 10) looked to have a key role in the self-assembly of collagen into fibrils, stabilising the fibrous collagen layer, and potentially impacting hMSCs activity [38].

**Figure 5:**
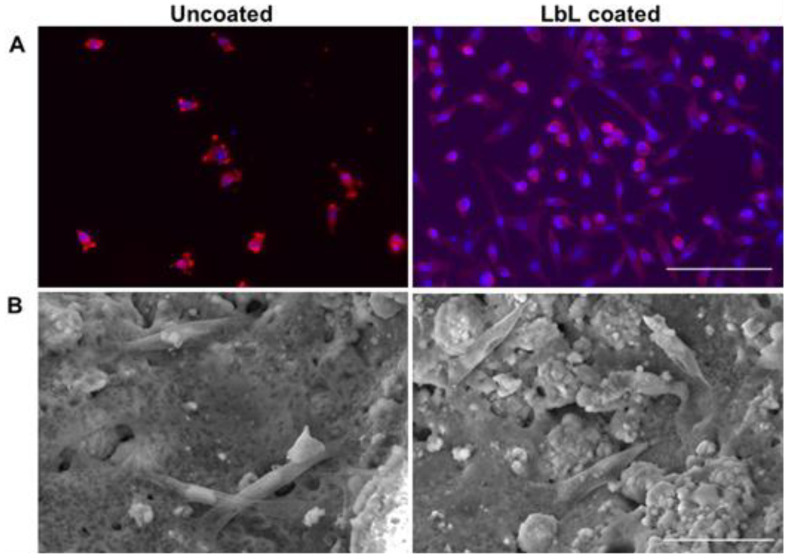
Y201 cells seeded on the porous scaffolds before (on the left) and after (on the right) Layer-by-Layer assembly functionalisation. (**A**) Immunostaining after 14 days with Nuclei in blue (DAPI) and cytoskeleton in red (PhRhod) (scale bar = 150 µm) and (**B**) SEM analysis after 21 days (scale bar = 50 µm).

In terms of cell morphology, the importance of the coating was observed in the immunostaining analysis where cells kept their spindle-shape on the coated samples, while on the uncoated scaffolds a lower number of adhered cells was observed and characterised by a more rounded shape and cellular contraction with smaller nucleus (Figure 5A).

It is normally recognised that Alkaline phosphatase activity changes are related with the differentiation state occurring in bone cells. Indeed, the increase of ALP activity is associated with new bone, increasing during the stages of bone formation [39]. In this work, the functionalisation with the LbL assembly of the glass-ceramics scaffolds allowed higher ALP activity, compared with the uncoated samples (at day 21 ALP expression of 0.040 ± 0.009 for the coated and 0.021 ± 0.006 for the uncoated, ** *p* < 0.01) (Figure 4B). However, higher activity for the uncoated samples was observed at 14 and 21 days of culture compared with the ALP expression at 7 days (* *p* < 0.05), showing that they were able to increase ALP expression during the differentiation process of Y201s to osteoblasts.

Along with the biological results described above, Y201s differentiation level was studied by quantitative expression of two important bone proteins, osteocalcin (OC) and osteopontin (OP). Figure 4C,D show the relative expression of those proteins normalised in respect to with the cell proliferation. It is largely reported in literature that osteoblasts are differentiated cells that mineralise bone matrix. Firstly, OP is a phosphoprotein, synthesised by bone forming cells, characterised by Ca binding domains and impacts cell adhesion, proliferation, and ECM mineralisation [40]. Whereas OC is a bone-specific glycoprotein able to bind Ca, which encourages the calcification of the new formed ECM [41]. As observed for the ALP expression, OP and OC assessment displayed at day 7 no significant difference in both protein expressions on the coated samples in respect to the uncoated scaffolds. However, at day 14 there it was noticed a significant increase in OP levels, which indicated the beginning of the mineralisation phase. (3465 ± 109 and 2598 ± 103 ng/μg DNA after and before the LbL assembly, * *p* < 0.05). To emphasise, the coated samples also revealed a significant OP overexpression at day 21, supporting the increased ALP activity. For OC assessment, there was a high protein expression up to day 21 (Figure 4D) for both type of scaffolds, indicating bone ECM maturation [41]. Notable is that the LbL-functionalised scaffolds showed a peak of expression already at day 14, suggesting that these scaffolds could induce OC protein expression in long term, that validates the ALP results.

From SEM analysis (Figure 5B), differences in the calcium deposition between the functionalised and unfunctionalised samples can be noticed: apatite can be observed in cauliflower form, which is usually the product of bioactivity [42].

## 4. Conclusions

Biomimetic glass-derived scaffolds coated with bioresorbable gelatin and chondroitin sulphate bio-resorb-able gelatin were prepared to mimic the bone native structure, consisting of collagen and natural mineral phase. The obtained functionalised coating was at the nanoscale, without affecting the total porosity and the resulting interconnectivity. The multilayered coating of gelatin and CS-DP improved significantly the Y201 growth of the porous glass ceramic scaffolds. The presence and high density of biomolecules (gelatin and CS-DP) in scaffolds, also stimulated the osteogenic activity, as demonstrated by the evaluation of OP and OC content after 21 days of cell culture. It was determined that the multilayered nanoscale coating using the LbL approach has confirmed the high potential to be used as innovative manufacturing technique to significantly enhance bone tissue regeneration when using appropriate orthopedic porous scaffolds as medical devices. Particularly, the LbL nanocoating can be suitable to incorporate biomolecules and/or drugs to: (1) enhance the antibacterial properties, i.e., with incorporation of antibiotics, silver nanoparticles, etc, to avoid the risk of nosocomial infections when the scaffolds are implanted in vivo, and (2) improve the formation of new bone, i.e., with the incorporation of BMP-2 growth factors, to accelerate the osteo-integration with the surrounding tissues in vivo.

## Figures and Tables

**Table 1:** Atomic concentration (%) of the characteristic elements present in the multilayer after Layer-by-layer assembly on the 3D porous glass-ceramic scaffolds.

Sample	C1s (%)	O1s (%)	N1s (%)	S2p (%)	S/N Ratio
1 Layer	73.4 ± 0.3	24.9 ± 0.2	1.6 ± 0.2	-	-
2 Layers	73.9 ± 0.5	25.1 ± 0.5	1.5 ± 0.1	0.4 ± 0.1	0.26 ± 0.13
3 Layers	73.2 ± 0.2	24.7 ± 0.6	1.8 ± 0.3	0.2 ± 0.1	0.11 ± 0.08
4 Layers	73.6 ± 0.4	24.2 ± 0.2	2.0 ± 0.3	0.7 ± 0.1	0.35 ± 0.17
5 Layers	73.2 ± 0.3	24.3 ± 0.1	1.9 ± 0.2	0.2 ± 0.1	0.10 ± 0.09
6 Layers	73.0 ± 0.8	24.2 ± 0.8	2.0 ± 0.7	0.8 ± 0.1	0.40 ± 0.07
7 Layers	73.3 ± 0.5	24.5 ± 0.2	1.9 ± 0.4	0.2 ± 0.1	0.10 ± 0.12
8 Layers	73.5 ± 0.3	23.7 ± 0.6	2.1 ± 0.8	0.7 ± 0.1	0.33 ± 0.11
